# Supplementary material for: The rs368698783 (G>A) Polymorphism Affecting LYAR Binding to the Aγ-Globin Gene Is Associated with High Fetal Hemoglobin (HbF) in β-Thalassemia Erythroid Precursor Cells Treated with HbF Inducers
Source: Int J Mol Sci. 2023 Jan 1;24(1):776. doi: 10.3390/ijms24010776 (PMC9821710; doi:10.3390/ijms24010776)
Supplement: Supplementary file 1 [file ijms-24-00776-s001.zip › ijms-2040835-supplementary.pdf]

Article

The rs368698783 (G>A) polymorphism affecting LYAR binding to the Aγ-globin gene is associated with high fetal hemoglobin (HbF) in β-thalassemia erythroid precursor cells treated with HbF inducers

Cristina Zuccato [1,2], Lucia Carmela Cosenza [1], Matteo Zurlo [1], Giulia Breveglieri [1], Nicoletta Bianchi [3], IIaria Lampronti [1,2], Jessica Gasparello [1], Chiara Scapoli [4], Monica Borgatti [1,2], Alessia Finotti [1,2,*] and Roberto Gambari [2]

[1] Section of Biochemistry and Molecular Biology, Department of Life Sciences and Biotechnology, , Ferrara University, 44121 Ferrara, Italy; cristina.zuccato@unife.it (C.Z.); luciacarmela.cosenza@unife.it (L.C.C.); matteo.zurlo@unife.it (M.Z.); giulia.breveglieri@unife.it (G.B.); ilaria.lampronti@unife.it (I.L.);
jessica.gasparello@unife.it (J.G.), monica.borgatti@unife.it (M.B.); alessia.finotti@unife.it (A.F.)
[2] Center 'Chiara Gemmo and Elio Zago' for the Research on Thalassemia, University of Ferrara, 44121 Ferrara, Italy; gam@unife.it (R.G.)
[3] Department of Translational Medicine and for Romagna, Ferrara University, 44121 Ferrara, Italy; nicoletta.bianchi@unife.it (N.B.)
[4] Section of Evolutionary Biology, Department of Life Sciences and Biotechnology, Ferrara University, 44121 Ferrara, Italy; chiara.scapoli@unife.it (C.S.)
* Correspondence: alessia.finotti@unife.it (A.F.); Tel.: +39-0532-974510

Supplementary Materials

Supplementary Methods

SM1. Recruitment of patients, ErPCs isolation and ErPCs induction.

The patients were recruited and the blood samples obtained according to the Declaration of Helsinki following specific approvals of the study by the Ethical Committees of Ferrara Hospital. All the participants to the NCT03877809 (A Personalized Medicine Approach for β-thalassemia Transfusion Dependent Patients: Testing SIROLIMUS in a First Pilot Clinical Trial) signed an informed consent on the basis of approvals of the Ethical Committee in charge of human studies at Arcispedale S. Anna, Ferrara (release of the approval: November 14, 2018). For ErPCs isolation and testing, the two-phase liquid culture procedure was employed as previously described8. After 7 days of a phase I culture, the non-adherent cells were harvested, washed and then cultured in phase II medium8 in absence or in presence of HbF inducers. Control uninduced cells are ErPCs cultured in parallel with induced cultures, but in the absence of the inducer. The solubilization reagent (MeOH/water 1:1, v/v) was unable to cause at the concentration used any changes in relative values and total amounts of hemoglobin production.

SM2. RNA extraction from erythroid precursor cells (ErPCs).

The total cellular RNA was extracted from ErPCs by using TRI Reagent® (Sigma-Aldrich, Saint Louis, Missouri, USA), following the manufacturer's instructions. The protocol used for extraction of ErPCs RNA employed 800 µl of TRI Reagent® for a dry pellet of 4-6 x 10^6 cells, then chloroform and isopropanol volumes during extraction was proportionally adjusted accordingly to the initial volume of TRI Reagent® used; the isolated RNA was washed once with cold 75% ethanol, dried and dissolved in 10-20 µl nuclease free water before use.

SM3. RT-qPCR analysis of expression of globin genes and LYAR.

For gene expression analysis 500 ng of total RNA was reverse transcribed by using the TaqMan® Reverse Transcription Reagents and random hexamers (Applied Biosystems, Life Technologies, Carlsbad, CA, USA). Quantitative real time PCR assay, to quantify the expression of the globin genes, was carried out using two different reaction mixtures, the first one containing γ-globin probe and primers, the second one containing GAPDH, RPL13, β-actin probes and primers. The primers and probes used are listed in Table 1.

Each reaction mixture contained 1x TaKaRa Ex Taq® DNA Polymerase (Takara Bio Inc., Shiga, Japan), 300 nM forward and reverse primers and the 200 nM probes (Integrated DNA Technologies, Castenaso, Italy). The assays were carried out using CFX96 Touch Real-Time PCR System (Bio-Rad, Hercules, California, USA). After an initial denaturation at 95°C for 1 min, the reactions were performed for 50 cycles (95°C for 15 sec, 60°C for 60 sec). Data were analyzed by employing the CFX manager software (Bio-Rad, Hercules, California, USA). To compare gene expression of each template amplified, the ΔΔCt method was used [58]. The Lyar Assay was ID Hs00215132_m1, TaqMan assay 20x, Applied Biosystems, Thermo Fischer Scientific, Waltham, MA, USA.

SM4. *HPLC analysis of hemoglobins.*

The ErPCs were centrifuged at 1200 rpm for 8 minutes and washed with PBS (Phosphate buffered saline). The pellets were then resuspended in a predefined volume of water for HPLC (Sigma-Aldrich, St. Louis, Missouri, USA). Then, three freeze/thaw cycles on dry ice were performed in order to lyse the cells and obtain the protein extracts. Hemoglobin analysis was performed by loading the protein extracts into a PolyCAT-A cation exchange column and then eluted in a sodium-chloride-BisTris-KCN aqueous mobile phase using HPLC Beckman Coulter instrument System Gold 126 Solvent Module-166 Detector which allows us to obtain a quantification of the hemoglobins present in the sample. The reading is performed at a wavelength of 415nm, and a commercial solution of purified human HbAF (Analytical Control Systems Inc., Fishers, IN, USA) extracts has been used as standard. The values thus obtained are processed using "32 Karat software". The % of HbF increase was identified by the following formula: 100 x (% HbF in induced cells - % HbF in control uninduced cells)/(100 - % HbF in control uninduced cells).

SM5. Statistical analysis.

All the data were normally distributed and presented as mean ± S.D. Statistical differences between groups were compared using one-way ANOVA (Analyses of variance) between groups. Statistical differences were considered significant when $p < 0.05$ (*), highly significant when $p < 0.01$ (**).

Supplementary Figures

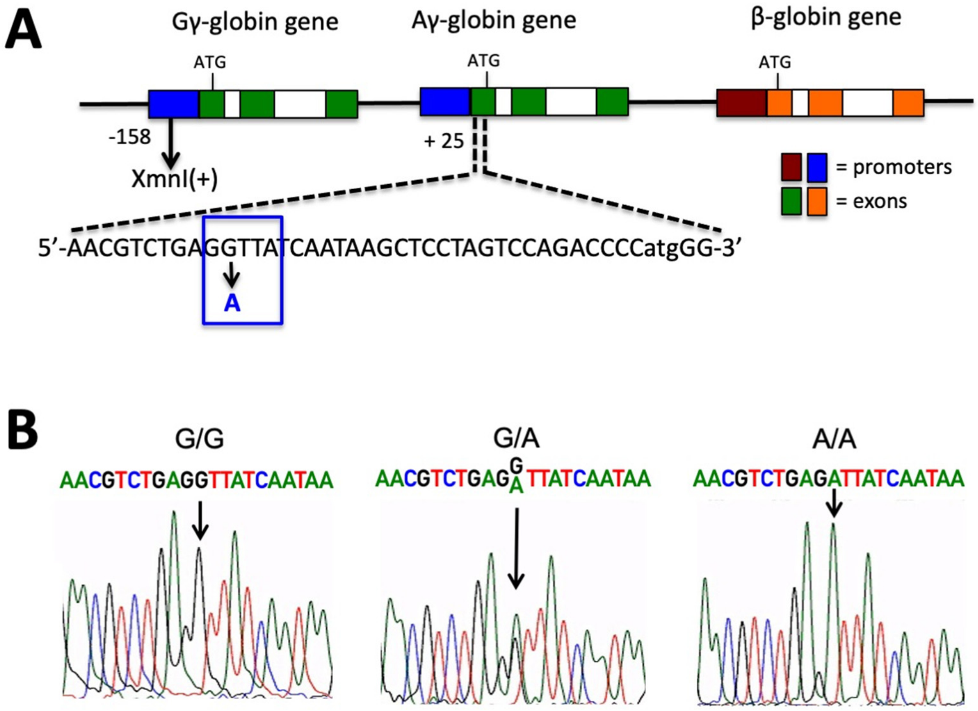

Figure S1. A. Location (A) and sequences (B) of the Aγ-globin gene rs368698783 (G>A) polymorphism. Extraction of genomic DNA and conditions for sequencing reactions are reported elsewhere [16]. The panels are modified from Breveglieri et al. [16], with permission (copyright can be found at https://www.ncbi.nlm.nih.gov/pmc/articles/PMC5575872/) [16].
